# Estimating willingness to pay for motorcycle helmet and its determinants through contingent valuation method

**Published:** 2024-07

**Authors:** Hadi Hosseini, Mina Golestani, Homayoun Sadeghi Bazargani, Mohammad Saadati

**Affiliations:** ^*a*^ Road Traffic Injury Research Center, Tabriz University of Medical Sceinces, Tabriz, Iran.; ^*b*^ Department of Public Health, Khoy University of Medical Sciences, Khoy, Iran.

**Keywords:** Willingness to pay (WTP), Motorcyclist, Safety helmet, Accidents

## Abstract

**Background:**

Helmet use has been introduced as one of the most highlighted strategies for preventing death and injury in motorcyclists. On the other hand, the cost of the helmet was introduced as a barrier to use. This study aimed at estimating the willingness to pay (WTP) of motorcyclists in Saqqez to buy and use safety helmets through the contingent valuation method (CVM).

**Methods:**

This cross-sectional study was conducted in 2021 in Saqqez County, Kurdistan Province, Iran. Sampling was performed by two-stage clustering through probability proportion to the size. The sample size was 570. A preliminary questionnaire was designed and administered for validity and reliability approval. Data analysis was carried out through linear regression models using STATA 15.

**Results:**

In total, 510 questionnaires were analyzed. The motorcyclists’ mean age was 31.37 years (SD = 8.48). Only 251 individuals (49.22%) had an appropriate license, and approximately 40% declared that they never used a safety helmet. The mean WTP was estimated to be 3900000 Rials (15/6 US$) through CVM. Motorcyclist age, helmet use by the motorcyclist friends, and socio-economic status were found to be significantly correlated with their WTP.

**Conclusions:**

The effect of socio-economic status on motorcyclist WTP highlights the significance of adopting supportive policies in this regard, along with stakeholder participation from the private sector and insurance companies. Moreover, the positive effect of helmet use by friends on individuals’ WTP shows that improving the general culture of traffic safety can be accompanied by promising results, especially for motorcyclists.

## Introduction

According to the World Health Organization (WHO), in 2023, with a slight reduction, nearly 1.19 million individuals lose their lives annually due to traffic accidents, and millions more are injured.^1^ Pedestrians, motorcyclists, and bicyclists, as vulnerable road users, account for nearly half of this figure.^1,2^ Motorcyclists are identified as users who are more likely to be involved in an accident. In comparison to car drivers, motorcyclists have an eight-fold higher risk of fatality, four times more injury risk, and two times higher risk of a collision with a pedestrian.^3-5^ Motorcyclists account for a disproportionate fraction of fatal incidents compared to other road users.^1,6,7^ This is due to their lack of protection, high speed, and refusal to wear helmets.^4,8,9^ Head trauma is the main fatality factor among motorcyclists.^10,11^ Given this concern, helmet wear promotion is crucial to motorcycle safety measures.^12-14^ The results of a systematic review indicated that wearing a helmet decreases the risk and severity of injury by up to 72% and likelihood of fatality by up to 39%, depending on the speed at which the motorcyclist was involved in an accident.^15,16^ This is despite the fact that the helmet use rate has been reported to be quite low, ranging from 10% and 50%, worldwide and the same in Iran.^16-19^ Sight limitation, discomfort, hafty helmet weight, and the helmet expense were illustrated as using barriers.^19,20^

Considering that motorcycles are used primarily by low socio-economic groups,^21,22^ helmet costs would be an uncoverable cost for them, which negatively affects their WTP. Willingness to pay is an approach for determining the highest preferred value that a person wants to pay to obtain a service or specific item.^23^ Regarding road traffic safety, this method has been commonly used to explore the value of statistical life (VSL), the monetary amount that a person is eager to pay to minimize injury or death risk.^24,25^ WTP of motorcyclists is reported to be affected by variables such as socio-economic status, education level, and accident history.^26,27^

The investigation of the various economic factors influencing the use of helmets can help policymakers in adopting particular initiatives to economically supporting the use of safety helmets. Most earlier studies employed direct approaches to assess willingness to pay.^24,28^ In these methods, individuals are asked to directly express their WTP. However, the direct questions about WTP are cognitively challenging assignments for the respondents, particularly about complex and unknown alternatives, which can lead to errors in the chosen option and artificial focus on the price.^29^ Furthermore, human beings do not necessarily have any reason for disclosing their WTP and they may probably display a greater or lower payout.^30,31^ Also, declaring their real valuation of a good or a policy might not be equivalent to a real choosing behavior.^30^ To address these limitations, the Contingent Valuation (CV) method was developed, which is based on the individuals stated-preferences to pay for a service or item.^32^ This study used the CV approach to estimate motorcyclists' willingness to pay for helmets and its determinants. 

## Methods 

Setting: This cross-sectional research was conducted during 2021 in Saqqez County, Kurdistan Province, Iran. Study Population and Sample: Motorcyclists from Saqqez city were selected as the study population. Two-stage cluster sampling was used for sampling as the city was divided into 5 clusters (north, west, centre, east, and south) based on municipal districting. Out of these five clusters, 3 were selected based on their census completeness by the health centre. According to information from the traffic police in Saqqez, there were 30,000 active motorcycles. Using Morgan's table, the study sample was estimated to be 380. Considering the design effect (i.e., 1.5), the study sample was expanded to 570 individuals. Sample allocation for each cluster was accomplished using the probability proportional to size (PPS) method (Table 1).

**Table 1:** Sample allocation based on clusters

Cluster	Population	Sample size
**Shahid Afshari Center**	18633	252
**Shahid Karbasi Center**	9813	133
**Shahid Mowludi Center**	13680	185

Sampling was carried out randomly through a street intercept survey^33^ on main streets and squares with substantial traffic volume in each cluster. The inclusion criteria were a minimum age of 18 years, consistent use of a motorcycle in the past three months, and domicile in Saqqez city. Motorcyclists who were passing through the city as travellers and riders under 18 years of age were excluded.

**Data Collection: ** The tool has three main sections: demographics, motorcycle riding behaviour, and a WTP questionnaire. Demographic information included of age, education level, marital status, self-reported socioeconomic status, and occupation. The second section encompasses the history and frequency of motor riding, reasons for motor riding, accident history within the last three months, possession of a motor riding license, frequency of helmet use, and reasons for helmet using/not using. A WTP questionnaire based on the CV method was developed through literature review and expert opinion, resulting in a list of questions and scenarios (item generation). To investigate the willingness to pay (WTP), a road crash scenario was developed. The respondents were asked to answer questions regarding their willingness to pay to evade the risk of death in the aforementioned scenario using two different question techniques as follows:

**Open-answer question: ** In this type of question, respondents were allowed to answer as they wished, meaning that individuals were interviewed without a limit set on the amount they were able and willing to pay.

**Bidimensional-dual questions: ** In this section, a number of questions were offered. First, the initial price (bid) was proposed based on the market price. The mean market price was determined based on actual market prices, which emerged through price acquisition from the local market. The second question was meant to inquire about the answer to the first question. If the answer to the first question was positive, the proposed bid would be doubled; otherwise, the price would be half. Due to the same rationale, questioning was conditioned in the bidimensional-dual method, resulting in responses that are more closely aligned with those of the real marketplace. This approach also gives individuals more opportunities to express their preferences during the bidding process.

The experts were given the items and asked to rate each one based on their relevance to the study aims, clarity, necessity, and simplicity in order to determine the validity of the questionnaire. Moreover, they were invited to express their thoughts on blending items, eliminating items, adding new items, or making any corrections to the contents of the items. Experts were required to have a minimum of 3 years of academic experience in the field of traffic safety or health economics, and a minimum of 5 years of experience in traffic police in order to meet the inclusion criteria. CVR and CVI coefficients were calculated based on expert opinions. A pilot research was carried out with 30 motorcycle riders from Saqqez to assess the questionnaire's reliability, and the Cronbach's alpha coefficient was somputed. The questionnaire's validity was confirmed by the experts (CVR=0.85, CVI=0.9), and its reliability was also confirmed (α=0.74).

**Data Analysis: **The calculation of CVI and CVR coefficients was carried out using Excel based on the following formula:

**formula:**
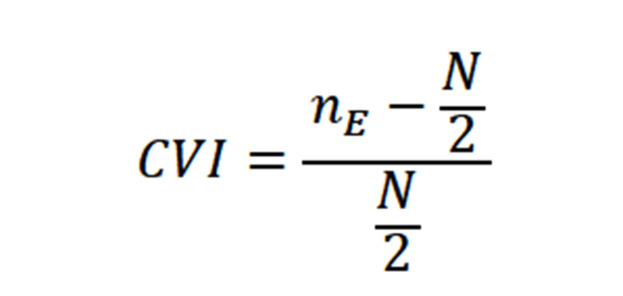
formula

Where nE is the number of experts who have provided answers to the option “necessary” and N is the total number of all the experts. The calculation of Cronbach’s alpha coefficient, as well, was conducted in SPSS Software.

Command doubled from the STATA15 software package was used to determin willingness to pay and also for double-bounded estimation. The Interval data model has been employed, which determines the willingness to pay based on the CVM method through the following hypothesis: 

1) If the individual should answer yes to the first question and no to the second question, t1<t<span=">2</t< <>; so, the following definition was obtained in this regard: t1≤WTP<t2 

2) If the addressee provides positive answers to both of the questions, the following formula should be followed: t2≤WTP<∞

3) If the individual answers no to the first question and yes to the second question, the following formula should be followed: <t2≤WTP<t1

4) If the individual answers no to both of the questions, the following formula should be followed: 0≤WTP<t<span="">2</t<>

The possibility of each of these four states would be as follows: 

1) yes, no: Pr (s, n) = Pr(T1≤WTP<t<span="">2)</t<>

2) yes, yes: Pr (s, s) = Pr(WTP>T1, t2≤WTP)

3) no, yes: Pr (n, s) = Pr (T2≤WTP<t< span="">1) </t<>

4) no, no: Pr (n, n) = Pr(WTP<t<span="">1,WTP<t<span="">2) </t<></t<>

In order to estimate beta and alpha, the maximum likelihood method was employed. We were requuired to determine the model's parameters in order to maximize the function. After estimating the model and obtaining the coefficients of the variables, using the average value of the explanatory variables, the mean willingness to pay was calculated. Finally, data processing yielded the following relationship: 

CI/ Mean = upper bound-lower

 bound Mean WTP= 

**formula:**
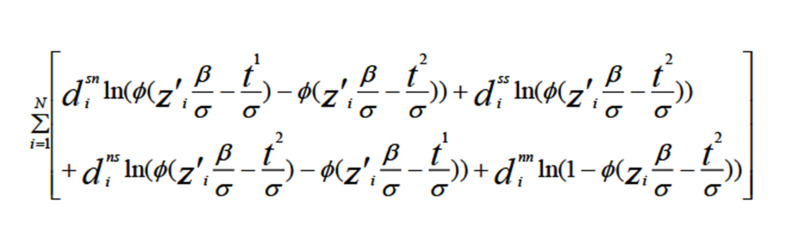
bound Mean WTP

d_i_^sn^,d_i_^ss^,d_i_^nd^ and d_i_^nn^ are variables that take a value ranging from zero to 1 according to each individual's answers. So, considering the above equation, δ and β were extracted, and the mean WTP was calculated which considered as the dependent variable. The analysis of the variables’ interrelationships was carried out using linear regression models. The logarithm transformation on the WTP was carried out as the distribution was skewed. All statistical analysis was done using STATA Software, version 15. 

## Results

A total of 510 questionnaires were analysed (response rate = 89.4%). The motorcyclists mean age was 31.37 (SD = 8.48) with a max and minimum of 54 and 18, respectively. Approximately 43% of participants (n = 216) were married. The demographic characteristics of the participants are presented in Table 2. 

**Table 2:** Description of the motorcyclists’ demographics

Variables		Number	Percentage
**Education**	Illiterate	17	3.33
	Under Diploma	98	19.22
	Diploma	228	44.90
	BSc	138	27.06
	MSc and higher	28	5.49
**Occupation**	Worker	126	24.71
	Employee	128	25.10
	Self-employed	198	38.82
	Other	58	16.86
**Social-economic status**	Very lower than the community mean	86	16.86
	Lower than the community mean	229	44.90
	Equal to the community mean	169	33.14
	Higher than the community mean	17	3.33
	Very higher than the community mean	9	1.76

The mean riding experience of the participants was 72.99 months (SD=35.83). The mean hour of daily motorcycle usage was 4.94 hours (SD=2.50), with a median of five hours per day. Among the motorcyclists surveyed, only 251 (49.22%) held valid motorcycle riding licenses, and 30% (153 individuals) had been involved in accidents during the previous three months. The majority of participants (291, 57.06%) cited daily errands as the main reason for riding motorcycles. Approximately 40% of the motorcyclists claimed that they never using a safety helmet, while only 9% reported always wearing one. Most of the individuals (55%) stated that they use the helmet for its safety benefits. Reduced visibility (27.18%) and expensive cost of helmets (29.6%) were among the most highlighted reasons for not using a helmet. 

The mean willingness to pay (WTP) for motorcyclists was calculated to be 3,900,000 Rials (equivalent to 15.6 US dollars at an exchange rate of 250,000 Rials). The results of participants' stated preferences regarding helmet prices have been presented in Table 3. 

**Table 3:** The status of responding to three prices proposed for safety helmet

Motorcyclists’ price acceptance status	First bid (200000 Rials) (n=510) Number (%)	Lower bid (100000)(n=151) Number (%)	Higher bid (400000) (n=359) Number (%)
Acceptance of the bid	359 (70.39%)	25 (16.56%)	248 (69.08%)
Non-acceptance of the bid	151 (29.61%)	123 (83.44%)	111 (30.92%)

In order to investigate the factors influencing the WTP of motorcyclists for purchasing a helmet, linear regression models were used. To accomplish this, univariate analysis was conducted initially. Next, significant variables at a 0.2 significance level were included in a multivariate model. The results of the univariate analysis of the factors affecting WTP have been presented in Table 4 . A significant association was found between age and WTP in a way that with each year increase in the age, the score of WTP has raised by 5.12. The use of helmets by motorcyclist friends was another variable that positively influenced the WTP level (P-value <0.001, β = 197.38). Ultimately, individuals with a lower socioeconomic status compared to the community mean were found to have a significantly lower WTP (P-value <0.001, β = -177.20).

**Table 4:** Univariate analysis of the determinants of WTP for helmet

Variable	Standardized Coefficient	95% CI	P-value
**Age**	5.12	0.3 to 9.92	0.037
**History of riding (Months)**	0.11	-1.01 to 1.23	0.851
**Riding duration per day (Hours)**	-14.29	-30.69 to 2.12	0.088
**Education (Academic)**	72.99	-14.29 to 160.93	0.104
**Marital status (Married)**	-1.96	-84.83 to 80.91	0.963
**Having license**	26.72	-55.03 to 108.47	0.522
**Job (Self-employed)**	4.80	-78.56 to 1.23	0.910
**Having a past accident experience**	10.33	-78.85 to 1.23	0.820
**Having insurance **	53.24	-33.97 to 140.45	0.231
**Engine’s volume (150cc and higher)**	-53.03	-138.30 to 32.25	0.223
**Using motorcycle for earning money**	19.84	-66.03 to 105.71	0.651
**Using motorcycle for doing the daily affairs**	77.51	-4.97 to 294.49	<0.001
**Helmet using by motorcyclists friends (often)**	197.38	100.27 to 294.49	<0.001
**A history of being fined by the police**	58.48	23.33 to 140.33	0.161
**Social-economic status below the community average**	-177.20	-263.27 to -91.13	<0.001

In order to estimate the final goodness of fit using the stepwise method, the factors that were significant at the 0.2 level were included in the model. The modelling results indicated that only socio-economic status below the community mean (P-value <0.001, β=-158.33) and helmet usage by motorcyclists' friends (P-value <0.001, β=175.28) were significantly correlated with WTP for purchasing a helmet. The results for the mentioned model are presented in Table 5. 

**Table 5:** Multivariate analysis of the determinants of WTP for helmet

Variable	Standardized Coefficient	CI 95%	p-value
**Constant**	442.59	517.40-367.78	<0.001
**Helmet using by motorcyclists friends**	175.28	79.95-270.6	<0.001
**Socio-economic status below the community average**	-158.33	-243.27 to -73.40	<0.001

## Discussion

The willingness to pay (WTP) of motorcyclists, based on the contingent valuation method, was determined to be 3,900,000 Rials (equal to 15.6 US dollars at an exchange rate of 250,000 Rials). The use of helmets by motorcyclist friends as well as theri socio-economic status was found to be significantly associated with individuals' WTP for helmets.

Evidence has shown that motorcycle usage was more common among individuals with lower levels of education and socio-economic status compared to other social classes.^21,22,34^ Our results similarly indicated that just 32% of the participating motorcyclists have earned academic education, and more than 60% of them reported having a low socio-economic standing. Almost half of the motorcyclists who took part in this study did not have a valid motorcycle riding license. According to the Mani et al. (2018) study in Shiraz, the results indicated that only 16.7% of motorcyclists hold a standard motorcycle riding license.^35^ Sotoodezadeh et al. (2021) also stated that the majority of young motorcyclists do not own riding licenses.^36^ Lacking a license means that the individual may not have the necessary skills and knowledge of motorcycle safety precautions, increasing the likelihood of injury or death. Moreover, they are unfamiliar with traffic rules, which cause to violations in the traffic environment. 

A study by Khanh H et al. (2008) in Vietnam found that households are willing to pay 9.38 USD for a helmet.^28^ Another study in Myanmar (2018) illustrated that the mean willingness to pay of motorcyclists for a 50% reduction in fatalities was $6.51, which is consistent with the results of our research.^25^ The variation in the willingness to pay amount may arise from the temporal and contextual differences in the studies. In contrast, Ainy et al. (2016) estimated that the motorcyclists WTP for minimizing injuries in Tehran was 888,110 Rials (equal to 35.52 US$), which is greater than the findings of our study.^24^ This could be related to the variation in the approach to estimating willingness to pay (WTP) and the study setting.

A significant association between motorcycle riders’ socioeconomic status and WTP emerged. The study by Naderi et al. (2018) in Urmia indicated that higher education and income enhance willingness to pay (WTP) for safety promotion.^26^ Chaturabong et al. (2011) also discovered in their study in Thailand that the WTP of motorcyclists for purchasing helmets and reducing accidents and injuries was significantly correlated with their income level.^27^ In a study conducted in Vietnam, the findings indicated that families with higher incomes were more willing to pay for reducing traffic risks.^28^ Similar findings have also been demonstrated by Mon et al. (2018) in Myanmar, indicating that the education and income level of individuals influence their willingness to pay.^25^ Higher income motivates individuals to invest in safety promotion. Given that motorcycles are typically utilized as a mode of transportation by low-income households and individuals, government support policies such as subsidies for purchasing helmets for motorcyclists can promote access to and encourage the usage of safety helmets.

Helmets using by the motorcyclists' companions was another factor influencing the WTP. Previous researches in Tehran and Kerman have also demonstrated the influence of friends on the adoption of safe behaviors and the usage or non-use of helmets by motorcyclists.^37,38^ Dong et al. (2018) illustrated a significant relationship between young people's driving behaviors and approval from friends.^39^ Similar results have also been reported in studies by Abdul Basit (2022) in Pakistan.^40^ These findings underscore the importance of peer influence in motorcycle safety promotion programs and initiatives. However, belonging to a same socio-economic status as the friend groups could be interpreted a basis for exhibiting similar behaviors.

## Conclusion

The willingness to pay (WTP) of motorcyclists, based on the contingent valuation method, was calculated to be 3,900,000 Rials (equal to 15.6 US dollars). Given the impact of socioeconomic status on WTP for helmets, it is recommended that supportive policies be adopted by the government to encourage helmet purchase in collaboration with other stakeholders, such as the private sector and insurance companies. Furthermore, the favorable impact of friends on willingness to pay (WTP) supports the idea that investing in promoting a general safety culture can yield positive effects, particularly for motorcyclists.

**Limitations: **This was a self-report study, like other ones, and may be due to over or underestimation. Moreover, in the generalization of the results, study setting characteristics should be considered. 

**Authors’ Contributions: **HH, MG, HSB and MS contributed to conceptualization, design and developing the protocol. HH, MG contributed to tool development. HH, MS and MG contributed to data collection and analyses. MS and HSB supervised all the work. HH, MG and MS prepared the draft and all the authors reviewed and approved the final version. 

**Acknowledgement**

The authors thank all the motorcyclists who took part in this study. They also would like to express their gratitude to the healthcare centers and traffic police in Saqqez County for their assistance in getting this research done.

## References

[1] World Health Organization. Global status report on road safety 2023. Geneva: World Health Organization; 2023.

[2] Saadati M, Razzaghi A, Rezapour R, PourEbrahim K (2022). Interventions for safety promotion of pedestrians; A scoping review. Journal of Transport & Health.

[3] Lin M-R, Kraus JF (2009). A review of risk factors and patterns of motorcycle injuries. Accid Anal Prev.

[4] Abedi L, Sadeghi-Bazargani H (2017). Epidemiological patterns and risk factors of motorcycle injuries in Iran and Eastern Mediterranean Region countries: a systematic review. Int J Inj Contr Saf Promot.

[5] Barros A, Amaral RL, Oliveira M, Lima SC, Gonçalves EV (2003). Traffic accidents resulting in injuries: underreporting, characteristics, and case fatality rate. Cad Saude Publica.

[6] Nickenig Vissoci JR, Krebs E, Meier B, Vieira IF, de Andrade L, Byiringiro F (2020). Road traffic crash experience among commercial motorcyclists in Kigali, Rwanda. Int J Inj Contr Saf Promot.

[7] Dhondt S, Macharis C, Terryn N, Van Malderen F, Putman K (2013). Health burden of road traffic accidents, an analysis of clinical data on disability and mortality exposure rates in Flanders and Brussels. Accid Anal Prev.

[8] El-Gabri D, Vissoci JRN, Meier BJ, Mvungi M, Haglund M, Swahn M (2020). Alcohol stigma as it relates to drinking behaviors and perceptions of drink drivers: A mixed method study in Moshi, Tanzania.. Alcohol.

[9] Tabary M, Ahmadi S, Amirzade-Iranaq MH, Shojaei M, Sohrabi Asl M, Ghodsi Z (2021). The effectiveness of different types of motorcycle helmets – A scoping review. Accid Anal Prev.

[10] Khodadadi N, Hosein Babaei Z, Charmi L, Alinia S, Asli A (2010). Epidmiology of trauma due to driving accidents in Poursina trauma research center in Rasht. Holistic Nursing and Midwifery Journal.

[11] Shahla A, Charehsaz S. Injuries resulting from motorcycle-induced trauma during two years in Shahid Motahari Clinical Center of URMIA. Iran J Forensic Med. 2006;12(2):79-83. (In Persian)

[12] Akbari M, Lankarani KB, Tabrizi R, Vali M, Heydari ST, Motevalian SA (2021). The effect of motorcycle safety campaign on helmet use: A systematic review and meta-analysis. IATSS Research.

[13] German CA, Soontornmon K, Singkham P, Tanasugarn L, Thienmongkol R, Weeranakin N (2019). A systematic review on epidemiology and promotion of motorcycle helmet use in Thailand. Asia Pac J Public Health.

[14] Papadopoulou J, Papakostopoulos V, Moulianitis VC (2021). Re-design of a motorcycle helmet for use in urban traffic: conceptual design and testing. Proceedings of the Design Society.

[15] Liu BC, Ivers R, Norton R, Boufous S, Blows S, Lo SK (2008). Helmets for preventing injury in motorcycle riders. Cochrane database of systematic reviews. 2008(1).. Cochrane Database Syst Rev.

[16] Keng S-H (2005). Helmet use and motorcycle fatalities in Taiwan. Accid Anal Prev.

[17] Mazloomy MahmoodAbad S, Mehri A, Morovati SharifAbad M, Fallahzadeh H (2007). Application of extended model of planned behavior in predicting helmet wearing among motorcyclist clerks in Yazd (2006).. J Birjand Univ of Med Sci.

[18] Baghianimoghadam MH, Zolghadr R, Ghafarzadeh J, Dashty M, Aram M (2010). A survey about attitude and practice of Yazd motorcycle drivers on using helmet. Toloo-E-Behdasht.

[19] Bazargani HS, Saadati M, Rezapour R, Abedi L (2017). Determinants and barriers of helmet use in Iranian motorcyclists: a systematic review. J Inj Violence Res.

[20] Haqverdi MQ, Seyedabrishami S, Groeger JA (2015). Identifying psychological and socio-economic factors affecting motorcycle helmet use. Accid Anal Prev.

[21] Nejatbakhsh EA, Akbari M, Bozorgi M. A study of the effects of monitoring motorcyclists on the reduction of inter-city accidents (a case study of Garmsar, 1386-1387). Traffic management studies. 2009;4(12):49-64. (in Persian)

[22] Torabi A, Tarahi M, Mahmoudi GA. Epidemiology of motorcycle accident in Khoramabad, Iran. Payesh. 2009;8(3):253-62. (in Persian)

[23] Champahom T, Banyong C, Hantanong N, Se C, Jomnonkwao S, Ratanavaraha V. Factors influencing the willingness to pay for motorcycle safety improvement: A structural equation modeling approach. Transportation Research Interdisciplinary Perspectives. 2023;22:100950.

[24] Ainy E, Soori H, Ganjali M, Basirat B, Haddadi M. Cost estimation of road traffic injuries among Iranian motorcyclists using the willingness to pay method. Arch Trauma Res. 2016 June; 5(2):e23198. 10.5812/atr.23198 PMC5035670 27679784

[25] Mon EE, Jomnonkwao S, Khampirat B, Satiennam W, Ratanavaraha V (2018). Willingness to pay for mortality risk reduction for traffic accidents in Myanmar. Accid Anal Prev.

[26] Naderi A, Fattahi S, Azami S. Estimating Willingness to Pay for Causality and Injury Reduction in Accidents Case Study: Urmia. Journal of Safety Promotion and Injury Prevention. 2018;6(3):152-162. (In Persian)

[27] Chaturabong P, Kanitpong K, Jiwattanakulpaisarn P (2011). Analysis of costs of motorcycle accidents in Thailand by willingness-to-pay method. Transportation Research Record.

[28] Pham KH, Le Thi QX, Petrie DJ, Adams J, Doran CM (2008). Households’ willingness to pay for a motorcycle helmet in Hanoi, Vietnam. Appl Health Econ Health Policy.

[29] Breidert C, Reutterer T (2007). Estimation of willingness-to-pay: Theory, measurement, application: Springer Science & Business Media; Deutscher Universitätsverlag Wiesbaden publication.

[30] Klingemann, W., Kim, JY., Füller, K.D. Willingness to Pay. In: Homburg, C., Klarmann, M., Vomberg, A. (eds) Handbook of Market Research. Springer, Cham.2022: 969–999.

[31] Davies HJ, Wu H, Schaafsma M (2023). Willingness-to-pay for urban ecosystem services provision under objective and subjective uncertainty. Resource and Energy Economics.

[32] Steigenberger C, Flatscher-Thoeni M, Siebert U, Leiter AM (2022). Determinants of willingness to pay for health services: a systematic review of contingent valuation studies. Eur J Health Econ.

[33] Buschmann A (2019). Conducting a street‐intercept survey in an authoritarian regime: the case of Myanmar. Social Science Quarterly.

[34] Dos Santos BH, Ahanhanzo YG, Kpozehouen A, Daddah D, Lagarde E, Coppieters Y (2021). Effect of wearing a helmet on the occurrence of head injuries in motorcycle riders in Benin: a case-control study. Inj Epidemiol.

[35] Mani A, Heydari ST, Sarikhani Y, Vossoughi M, Lankarani KB. Attention Deficit Hyperactivity Disorder as a determinant of motorcycle accidents in Fars province of Iran. J Inj Violence Res. 2019 Aug; 11(4 Suppl 2): Paper No. 22.

[36] Setoodehzadeh F, Moghadam AA, Okati-Aliabad H, Khammarnia M, Mohammadi M (2021). Self-reported Motorcycle Riding Behavior in Southeast of Iran. Health Scope.

[37] Maghsoudi A, Boostani D, Rafeiee M (2018). Investigation of the reasons for not using helmet among motorcyclists in Kerman, Iran. Int J Inj Contr Saf Promot.

[38] Zamani-Alavijeh F, Bazargan M, Shafiei A, Bazargan-Hejazi S (2011). The frequency and predictors of helmet use among Iranian motorcyclists: A quantitative and qualitative study. Accid Anal Prev.

[39] Duong HT, Parker L (2018). Going with the flow: Young motorcyclists’ misperceived norms and motorcycle speeding behaviour. Journal of Social Marketing.

[40] Basit HA, Khattak A, ABBAS Q, Abbas SA, Hussain A (2022). Assessment of Risk-Taking Behaviour of Young Motorcyclists at Un-Signalised Intersections–A Partial Least Square Structural Equation Modelling Approach. Promet-Traffic & Transportation.

